# Supplementary material for: Conformational dynamics and membrane insertion mechanism of B4GALNT1 in ganglioside synthesis
Source: Nat Commun. 2025 Jul 1;16:5442. doi: 10.1038/s41467-025-60593-9 (PMC12217166; doi:10.1038/s41467-025-60593-9)
Supplement: Supplementary file 1 — Supplementary Information [file 41467_2025_60593_MOESM1_ESM.pdf]

Supplementary Information for:

Conformational Dynamics and Membrane Insertion Mechanism of B4GALNT1 in Ganglioside Synthesis

This file contains:

Supplementary Figures S1-S11

Supplementary Table S1

Supplementary References

Additional files:

Supplementary Movie M1

Supplementary Primer Sequences

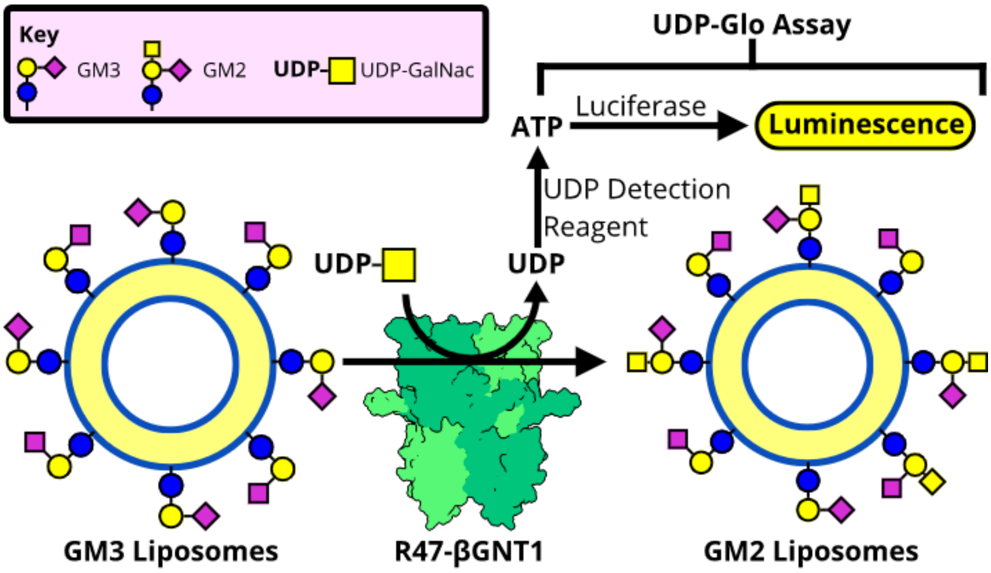

Supplementary Figure S1. Schematic diagram of the two-step B4GALNT1 activity assay. The UDP produced by B4GALNT1 following transfer of the GalNac group from UDP-GalNac donor substrate to GM3 lipid substrate is quantified using a UDP-Glo assay. Liberated UDP is converted to ATP and used as a substrate for luciferase producing a product detectable by luminescence.

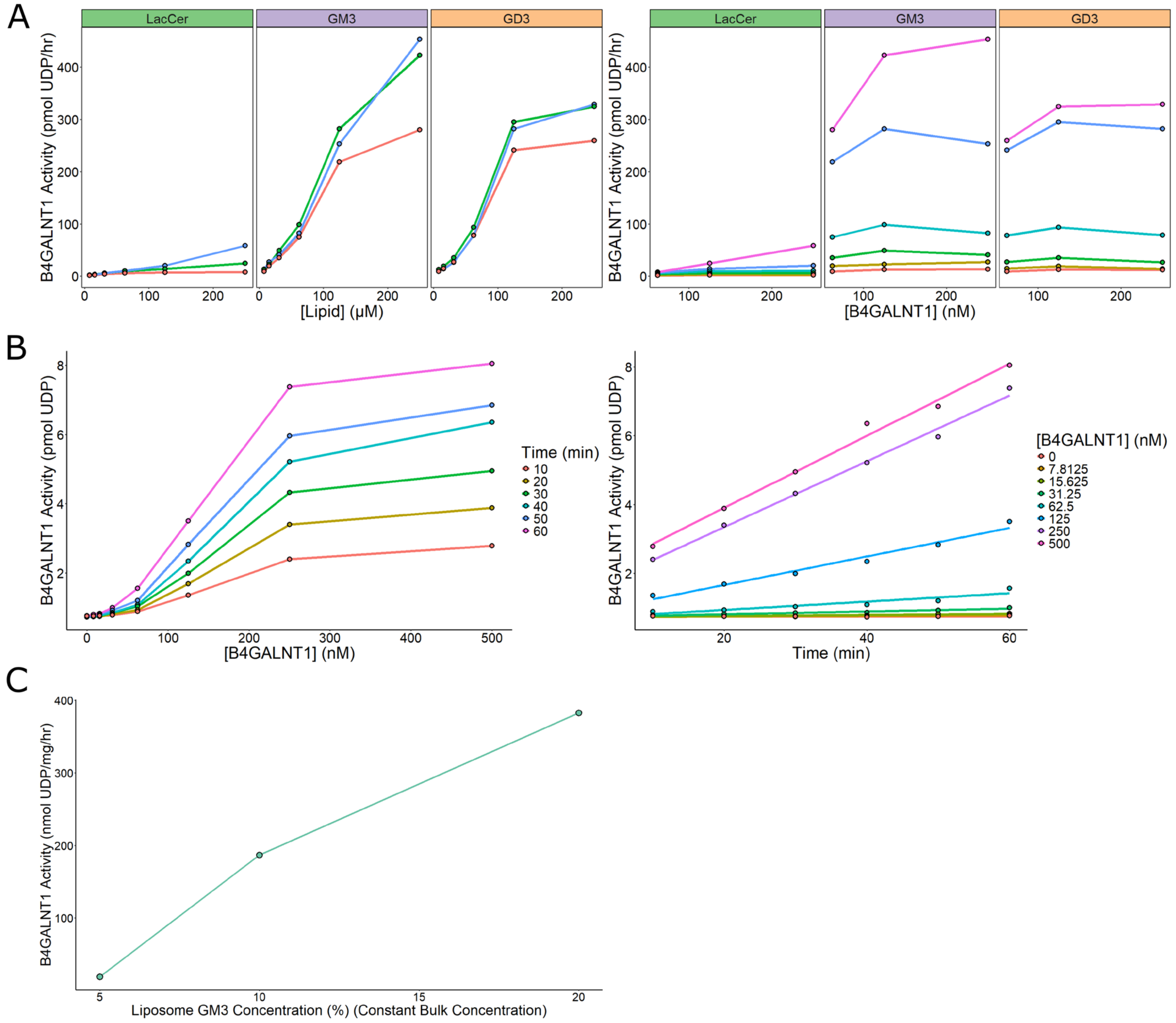

Supplementary Figure S2. Optimisation of B4GALNT1 activity assay conditions. (A) B4GALNT1 activity assays performed across different lipid (left panel) and enzyme (right panel) concentrations. **(B)** B4GALNT1 time-course activity assays across a range of enzyme concentrations using 1mM Sialyllactose as the acceptor substrate. In both (A) and (B) The UDP-Glo^{TM} assay system was used, with a UDP standard curve in order to calculate the concentration of UDP produced. **(C)** B4GALNT1 activity assay maintaining a constant bulk concentration of GM3 (500 μM), whilst reducing its surface concentration by increasing Phosphatidylcholine content in the liposome. His-tagged B4GALNT1 was recruited to the surface of the liposome via association with Ni-headgroup lipids (DGS-NTA(Ni)).

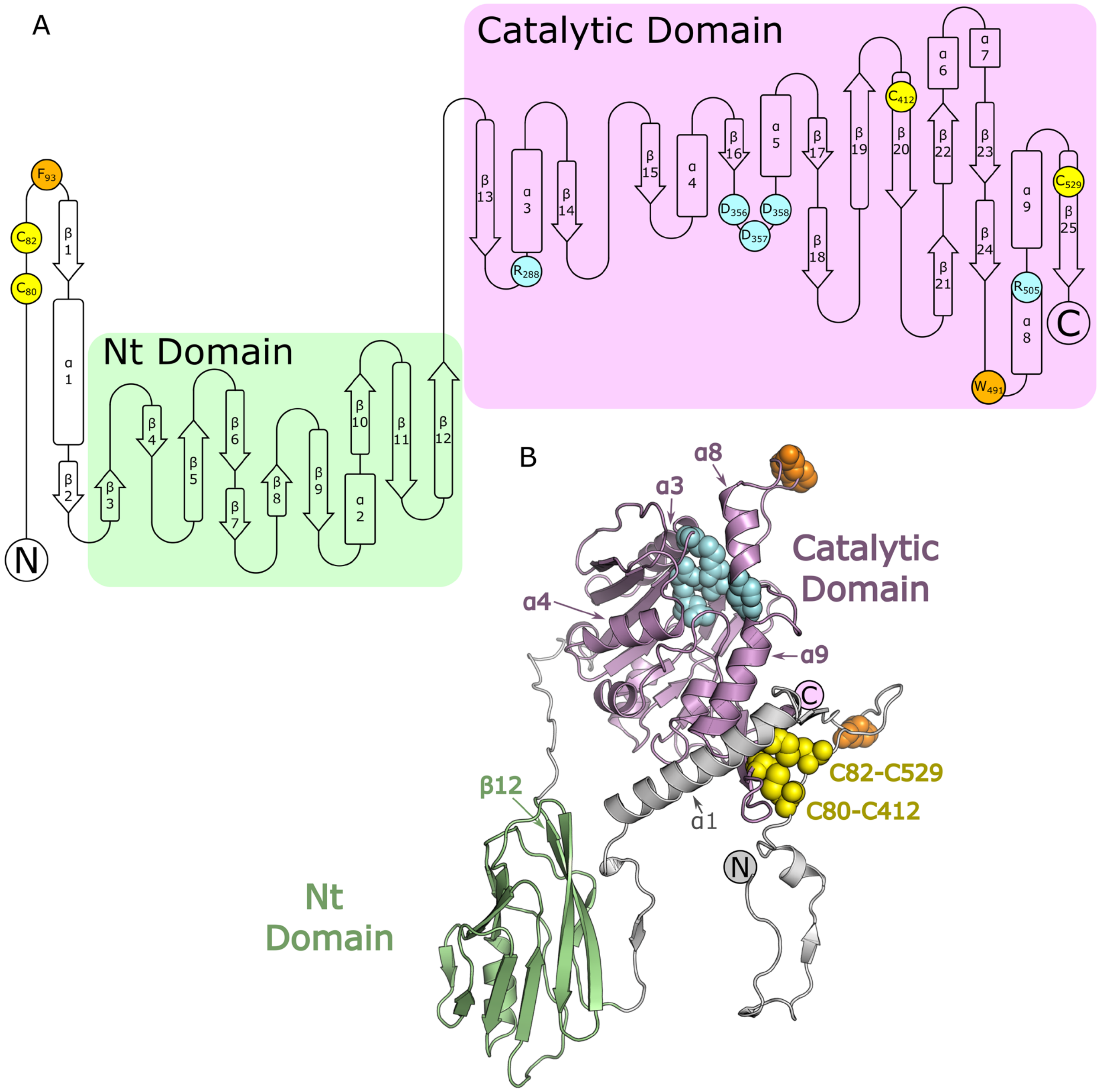

Supplementary Figure S3. Structural organisation of the B4GALNT1 lumenal domain. **(A)** Secondary structure diagram of a single chain of B4GALNT1 labelling α-helices and β-strands. Not all automatically annotated secondary structure elements were included and labelled in the diagram (very short secondary structure annotations were omitted). **(B)** 3D structure of one chain of the B4GALNT1 dimer. In both panels, the N-terminal (Nt) domain is highlighted in green and catalytic domain in purple. The cysteine residues involved in the disulphide bonds that anchor the N-terminus of the protein to the catalytic domain are highlighted in yellow. Catalytic residues are highlighted in cyan, and key membrane association residues are highlighted in orange. Secondary structural elements are labelled in (A) with a selection also highlighted in the structure (B). Details of the start and end residues for each structural element are provided in Supp. Table 1.

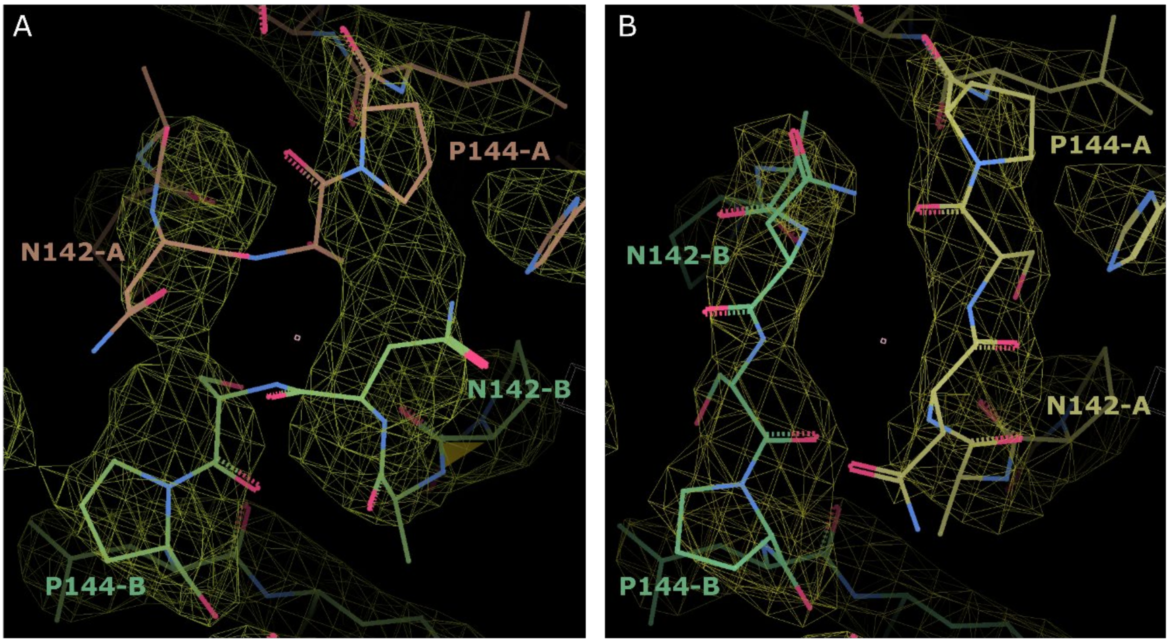

Supplementary Figure S4. Electron density maps demonstrating rethreading of the B4GALNT1 mainchain in the AF2 model versus the experimental crystal structure. (A) The original AF2 model had chain A (orange sticks) and chain B (green sticks) crossing over at residues N142-P144. Experimental $2F_0$-F_c electron density maps are shown as a yellow mesh. **(B)** Colouring as for (A) demonstrates that rethreading of these chains matches the electron density in the experimental structure of B4GALNT1.

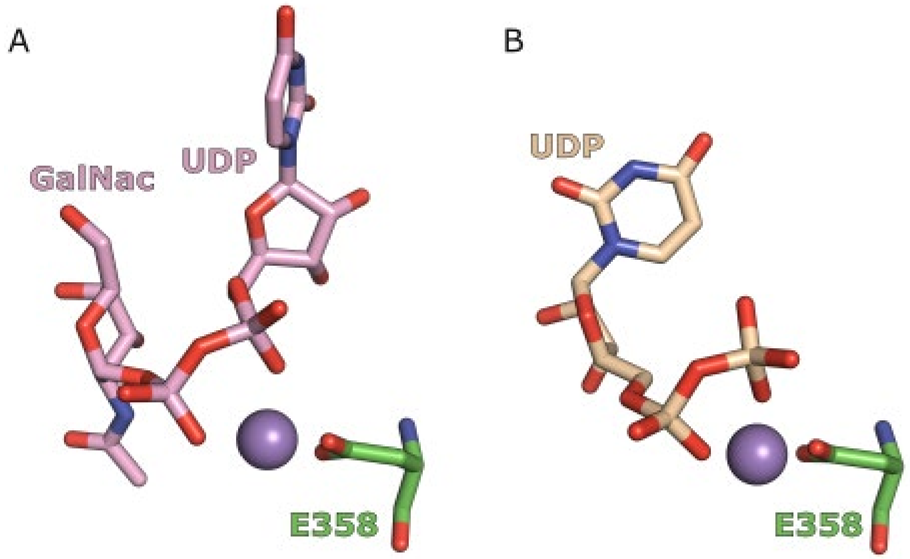

Supplementary Figure S5. Comparison of UDP substrate and product conformations in the B4GALNT1 active site. **(A)** The orientation of UDP-GalNac in the substrate complex is shown (pink sticks) relative to the Mn ion (purple) and residue E358 (green sticks) in the active site. **(B)** Orientation as for (A) but showing the UDP product (wheat sticks) in the active site. The UDP moiety is bound in an alternative conformation compared to the UDP in the UDP-GalNac-bound structure.

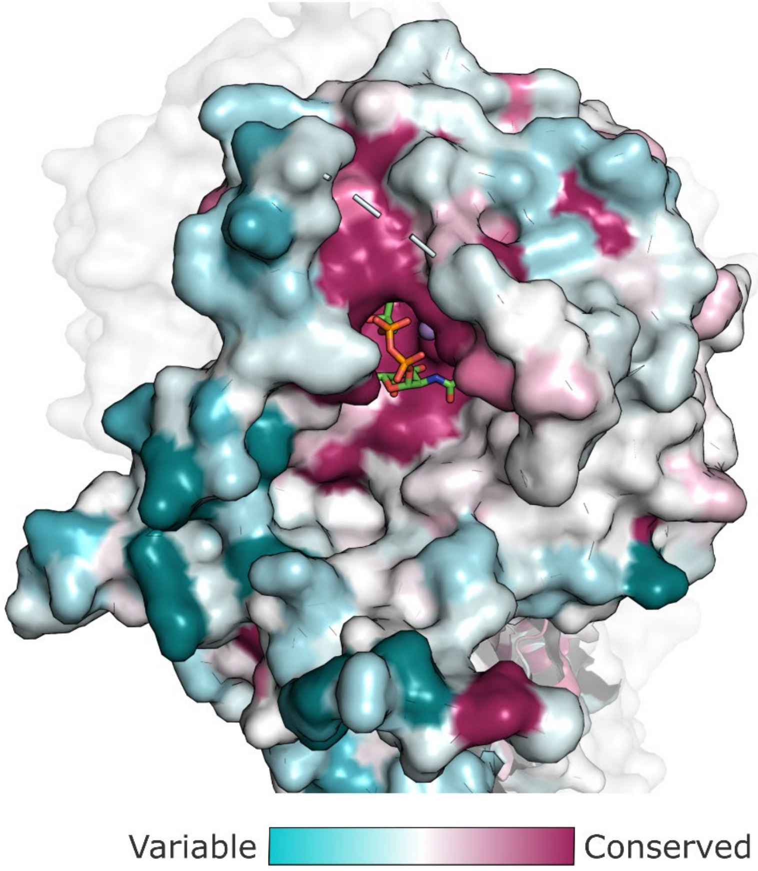

Supplementary Figure S6. Surface representation of the B4GALNT1 ligand binding site illustrating the UDP-GalNac (sticks) and showing conservation of residues across glycosyltransferase proteins. Sequence conservation was calculated and mapped onto the structure using Consurf [1] with colouring from highly variable (cyan) to highly conserved (magenta).

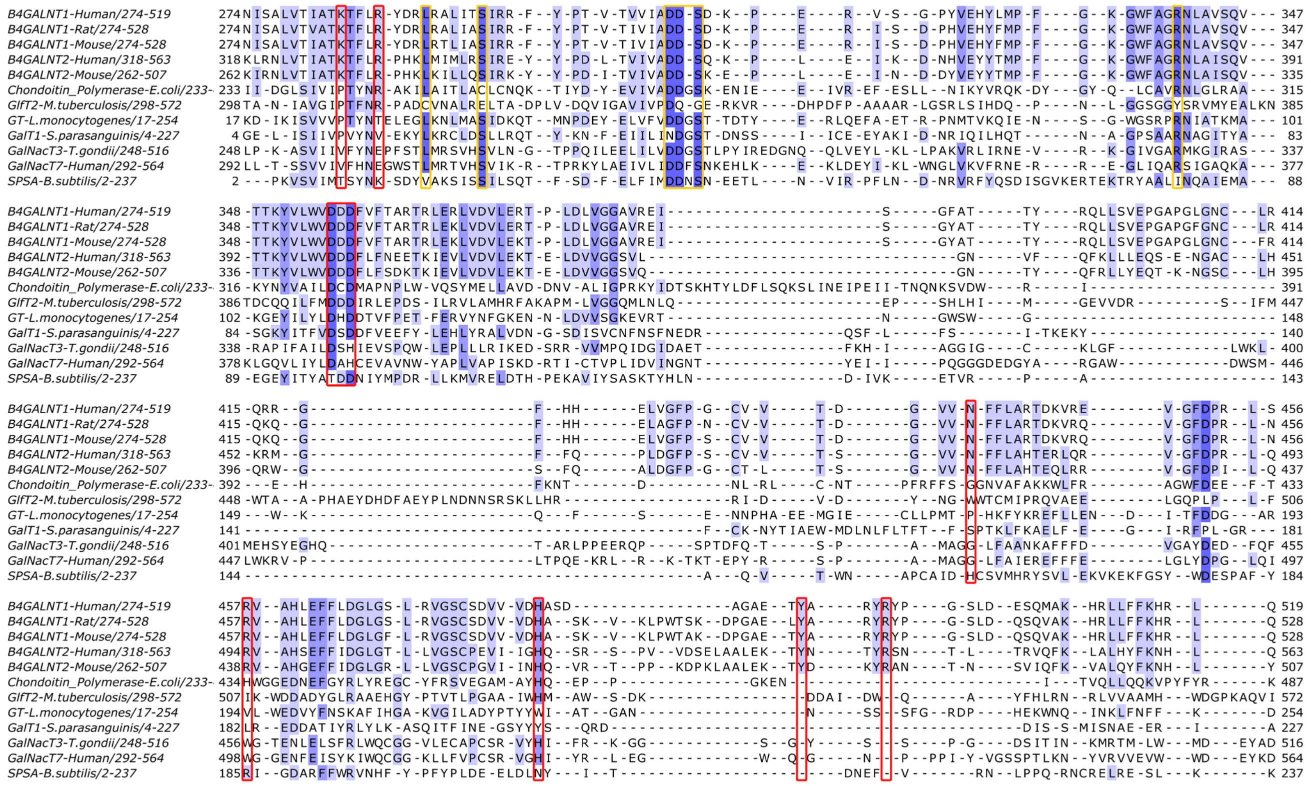

Supplementary Figure 7. Structure-based sequence alignment of the catalytic domain of B4GALNT1, the catalytic domains of AlphaFold models for CAZY family GT12 and the top 7 ranked hits from the DALI structural alignment webserver [2] following a search with the B4GALNT1 catalytic domain against the PDB25. The structural alignment was performed in VMD [3] using the Stamp Structural Alignment tool [4] and visualised using JalView [5]. Residues are coloured by percentage identity across the alignment (blue) and residues identified as important for UDP-GalNac binding are boxed in red. Additional highly conserved residues that may be important for stabilising structural elements around the active site are boxed in yellow.

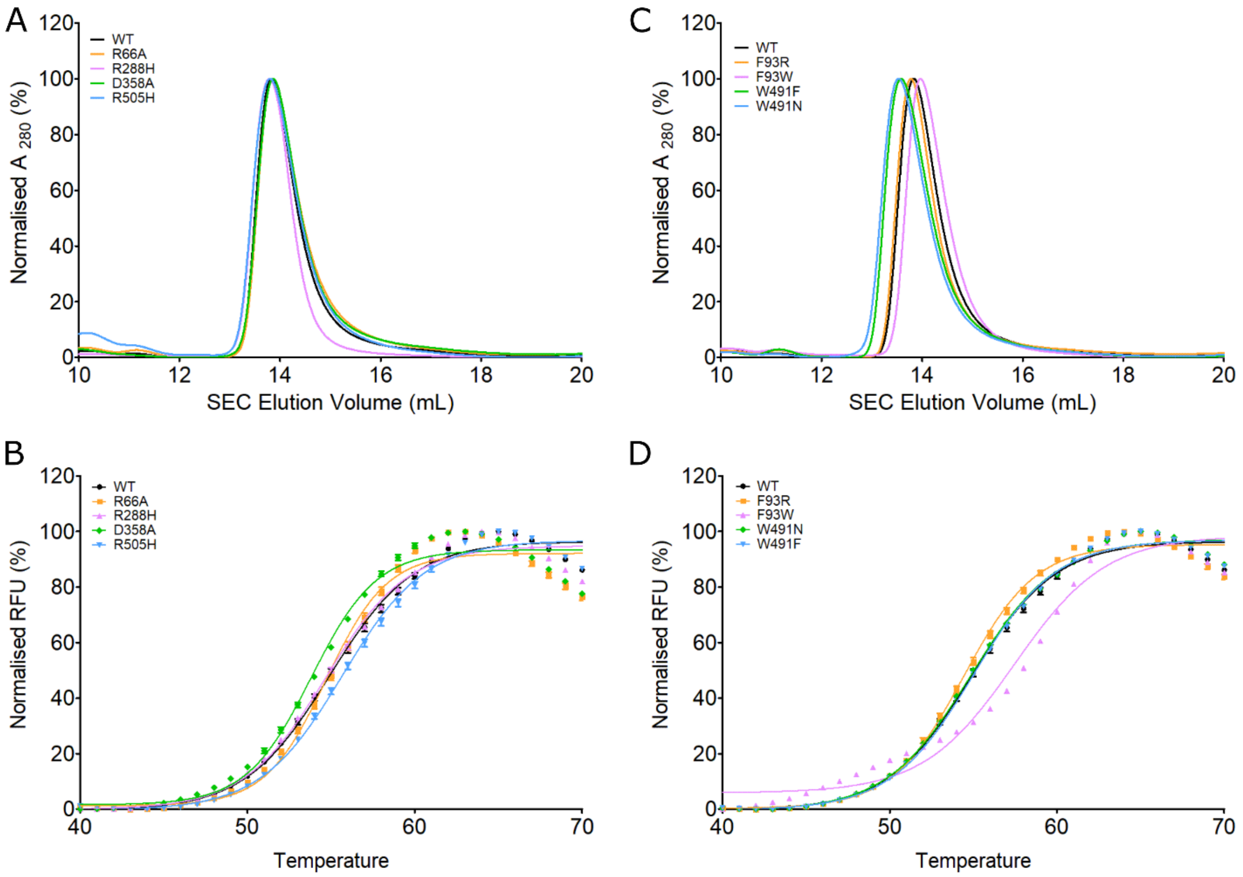

Supplementary Figure S8. Characterisation of B4GALNT1 mutants. **(A)** Elution profile following size-exclusion chromatography (SEC) for catalytic and disease mutants R66A, R288H, D358A and R505H. **(B)** Differential scanning fluorimetry (DSF) to monitor melting temperature as a measure of correct folding for the mutants as in (A). **(C)** SEC elution profiles for loop mutants F93R, F93W, W491F and W491N. **(D)** DSF melt curves for the loop mutants s in (C). Melt curves were carried out as N=1 for each mutant.

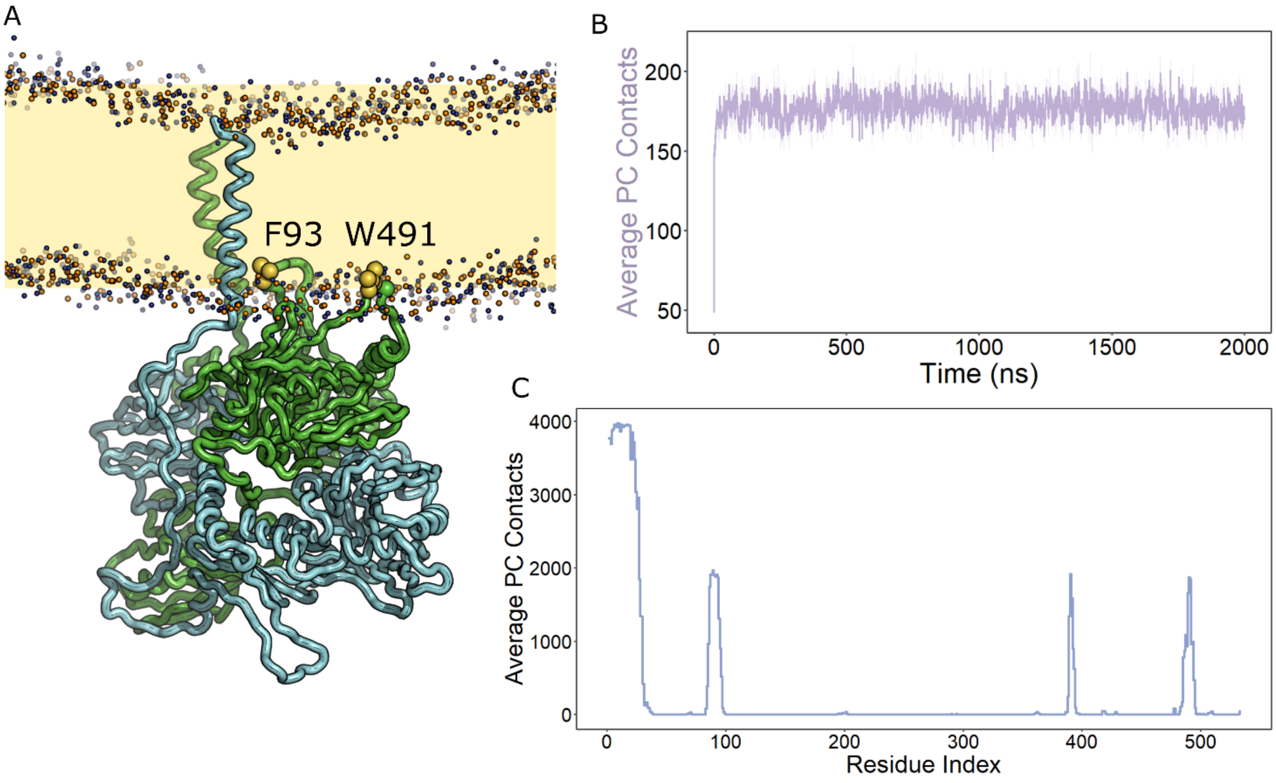

Supplementary Figure S9. Course-grain molecular dynamics simulations (MDS) for B4GALNT1 including the N-terminal transmembrane helix. **(A)** Illustration of a final pose of the full-length B4GALNT1 dimer in a PC membrane. As for the MDS with the luminal domain alone, the orientation of the lumenal domain consistently resulted in the insertion of the F93 and W491 loops of one chain of the dimer into the PC membrane. **(B)** Per residue analysis of the lipid contacts for the membrane-tethered B4GALNT1 reveal a similar pattern of luminal domain interactions with the membrane in addition to the transmembrane helix.

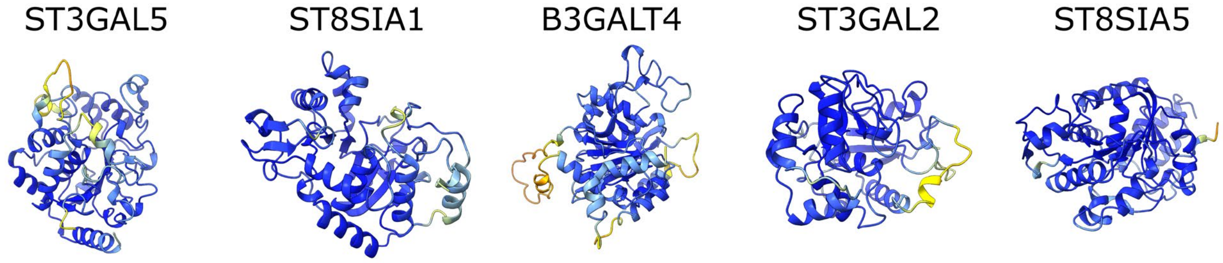

Supplementary Figure S10. Ribbon diagrams of AF2 predicted structures of the luminal domains of ganglioside synthetic enzymes coloured by pLDDT score. Colouring is from high confidence to low confidence prediction (blue through yellow to red).

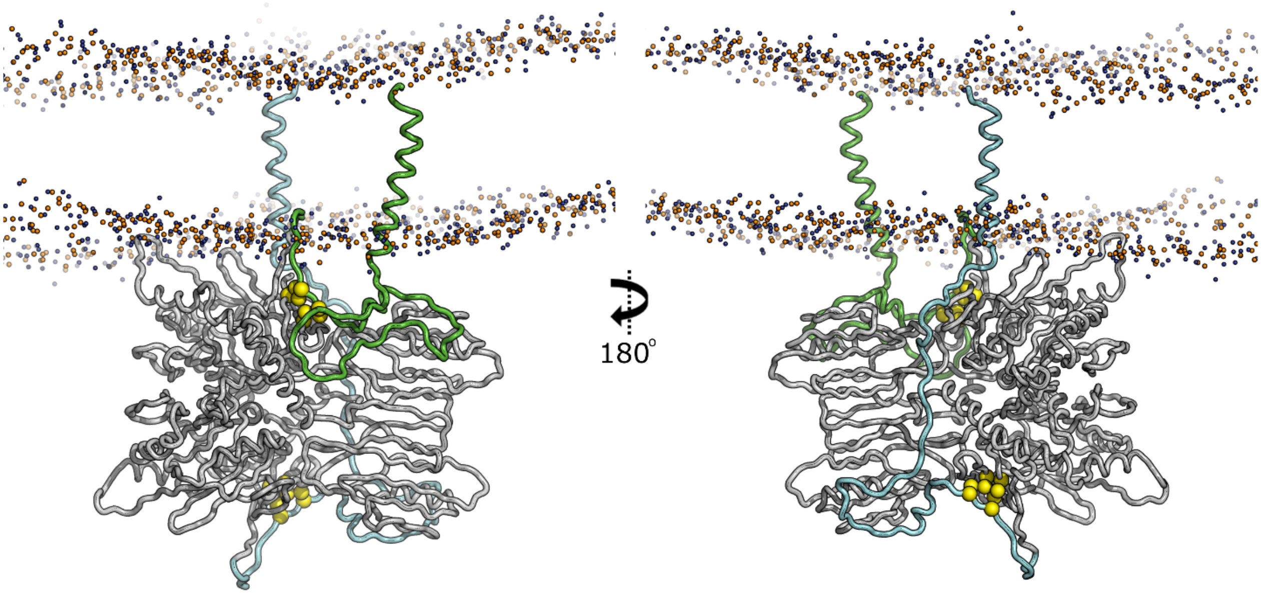

Supplementary Figure S11. Example poses of the full-length B4GALNT1 following MDS. For one molecule of the dimer the residues from the transmembrane helix to the N-terminal region of the active site (green) are not extended (left panel). This arrangement means that this region of the other molecule in the dimer (blue) is extended (right panel). This part of the molecule now spans from the membrane across the dimer and to the N-terminal region of the active site. The disulfide bonds that hold the N-terminal loop containing F93 are shown (yellow spheres).

Supplementary Table S1. Structural Location of B4GALNT1 Sequence Variants Identified in Patients with Spastic Paraplegia in the ClinVar Database [6]

Structural Element*	Start Residue	End Residue	ClinVar Missense Mutations Associated with Spastic Paraplegia **
Cytoplasmic tail	1	7	Trp2Leu, Arg5His, Arg5Leu, Ala7Thr
Transmembrane helix	8	25	Ala10Ser, Ala16Ser, Leu23Val
Juxtamembrane disordered loop	26	50	Thr27Asn, Leu33Pro, Pro36Leu, Pro36Ser, Arg47Cys, Pro64Arg, Gly73Arg, Asn79Ser, Cys82Tyr, Ser84Cys, Ser85Ile, Gly86Arg, Gly86Trp, Gly87Glu, Gly88Asp
Ordered N-terminal loop	51	97	Pro64Arg, Gly73Arg, Asn79Ser, Cys82Tyr, Ser84Cys, Ser85Ile, Gly86Arg, Gly86Trp, Gly87Glu, Gly88Asp
β-Strand 1	98	101	
Turn/Loop	102	107	
α-Helix 1	108	129	Pro108Leu, Ala109Glu, Ala114Pro, Glu119Asp, Ser127Leu
Turn/Loop	130	136	Ser131Pro
β-Strand 2	137	139	
Turn/Loop	140	146	Pro144Leu
β-Strand 3	147	150	Tyr147Cys
Turn/Loop	151	158	Val154Ile, Pro156Ser
β-Strand 4	159	161	
Turn/Loop	162	175	Gln168Arg, Gly172Ser
β-Strand 5	176	181	
Turn/Loop	182	184	
β-Strand 6	185	189	
Turn/Loop	190	190	
β-Strand 7	191	193	
Turn/Loop	194	196	
β-Strand 8	197	199	Thr197Ala, Leu198Phe
Turn/Loop	200	204	Glu201Gln
β-Strand 9	205	210	Thr207Ile
Turn/Loop	211	212	
α-Helix 2	213	222	Gln215Arg
Turn/Loop	223	222	
β-Strand 0	223	227	
Turn/Loop	228	232	Arg228Gln
β-Strand 11	233	242	
Turn/Loop	243	244	Glu243Gly
β-Strand 12	245	254	Arg252Ser, Arg252Cys, Ile253Val
Turn/Loop	255	278	Pro256Leu, Arg260Gly, Leu267Pro, Gln269His, Ala277Gly, Ala277Thr
β-Strand 13	279	285	
Turn/Loop	286	288	Arg288His
α-Helix 3	289	300	Tyr289Phe, Arg291Trp, Arg293Trp, Arg300His
Turn/Loop	301	306	Thr305Met

β-Strand 14	307	312	Ala311Thr
Turn/Loop	313	324	Asp315Asn, Val320Ile
β-Strand 15	325	329	
Turn/Loop	330	335	Pro331Ser, Gly333Ser
α-Helix 4	336	346	Trp336Cys
Turn/Loop	347	350	Lys350Glu
β-Strand 16	351	354	Tyr351Ser
Turn/Loop	355	366	Asp357Glu, Asp357His, Thr362Met, Ala363Gly
α-Helix 5	367	376	Glu375Ala
Turn/Loop	377	380	
β-Strand 17	381	383	Gly383Arg
Turn/Loop	384	384	
β-Strand 18	385	388	
Turn/Loop	389	397	Ala393Val, Arg397Pro, Arg397Trp
β-Strand 19	398	403	Ser401Arg, Val402Leu, Val402Met
Turn/Loop	404	410	Pro404Ser, Ala406Asp
β-Strand 20	411	417	
Turn/Loop	418	428	
β-Strand 21	429	432	
Turn/Loop	433	438	Asp433Ala
β-Strand 22	439	442	Phe439Leu, Ala441Glu
Turn/Loop	443	442	
α-Helix 6	443	449	
Turn/Loop	450	460	His460Tyr
α-Helix 7	461	466	Phe463Leu, Phe464Leu
Turn/Loop	467	472	Gly467Glu, Arg472Pro
β-Strand 23	473	476	
Turn/Loop	477	479	Asp478Asn, Val479Ile
β-Strand 24	480	483	Val480Met, Val480Leu
Turn/Loop	484	495	
α-Helix 8	496	505	Arg505His, Arg505Cys
Turn/Loop	506	511	Asp511Glu
α-Helix 9	512	525	Gln514Arg, Met515Ile
Turn/Loop	526	526	
β-Strand 25	527	533	

* Structural elements are annotated schematically and in 3D in Figure S3.

** Text colour indicates ClinVar pathogenicity designation. Black-Uncertain significance, Orange-Likely pathogenic, Red-Pathogenic.

SUPPLEMENTARY REFERENCES

1. Yariv, B. *et al.* Using evolutionary data to make sense of macromolecules with a 'face-lifted' ConSurf. *Protein Sci. Publ. Protein Soc.* **32**, e4582 (2023).
2. Holm, L. Dali server: structural unification of protein families. *Nucleic Acids Res.* **50**, W210–W215 (2022).
3. Humphrey, W., Dalke, A. & Schulten, K. VMD: visual molecular dynamics. *J. Mol. Graph.* **14**, 33–38, 27–28 (1996).
4. Russell, R. B. & Barton, G. J. Multiple protein sequence alignment from tertiary structure comparison: assignment of global and residue confidence levels. *Proteins* **14**, (1992).
5. Waterhouse, A. M., Procter, J. B., Martin, D. M., Clamp, M. & Barton, G. J. Jalview Version 2--a multiple sequence alignment editor and analysis workbench. *Bioinforma. Oxf. Engl.* **25**, (2009).
6. Landrum, M. J. *et al.* ClinVar: improvements to accessing data. *Nucleic Acids Res.* **48**, D835–D844 (2020).
