# Supplementary material for: Conformational dynamics and membrane insertion mechanism of B4GALNT1 in ganglioside synthesis
Source: Nat Commun. 2025 Jul 1;16:5442. doi: 10.1038/s41467-025-60593-9 (PMC12217166; doi:10.1038/s41467-025-60593-9)
Supplement: Supplementary file 2 — Description of Additional Supplementary Files [file 41467_2025_60593_MOESM2_ESM.pdf]

Description of Additional Supplementary Files

File Name: Supplementary Movie 1
Description: A video showing the simulation trajectory for a representative B4GALNT1 atomistic simulation with GM3 in a PC membrane. B4GALNT1 is coloured in green, GM3 in cyan and PC in beige. Video was rendered using VMD
